# *Itga2b* Regulation at the Onset of Definitive Hematopoiesis and Commitment to Differentiation

**DOI:** 10.1371/journal.pone.0043300

**Published:** 2012-08-28

**Authors:** Stephanie Dumon, David S. Walton, Giacomo Volpe, Nicola Wilson, Emilie Dassé, Walter Del Pozzo, Josette-Renee Landry, Bryan Turner, Laura P. O’Neill, Berthold Göttgens, Jon Frampton

**Affiliations:** 1 Institute for Biomedical Research, College of Medical and Dental Sciences, University of Birmingham, Birmingham, United Kingdom; 2 University of Cambridge Department of Haematology, Cambridge Institute for Medical Research, Cambridge, United Kingdom; 3 Institut de recherches cliniques de Montréal, Montréal, Canada; 4 Nikhef, Science Park, Amsterdam, The Netherlands; Emory University, United States of America

## Abstract

Product of the *Itga2b* gene, CD41 contributes to hematopoietic stem cell (HSC) and megakaryocyte/platelet functions. CD41 expression marks the onset of definitive hematopoiesis in the embryo where it participates in regulating the numbers of multipotential progenitors. Key to platelet aggregation, CD41 expression also characterises their precursor, the megakaryocyte, and is specifically up regulated during megakaryopoiesis. Though phenotypically unique, megakaryocytes and HSC share numerous features, including key transcription factors, which could indicate common sub-regulatory networks. In these respects, *Itga2b* can serve as a paradigm to study features of both developmental-stage and HSC- versus megakaryocyte-specific regulations. By comparing different cellular contexts, we highlight a mechanism by which internal promoters participate in *Itga2b* regulation. A developmental process connects epigenetic regulation and promoter switching leading to CD41 expression in HSC. Interestingly, a similar process can be observed at the *Mpl* locus, which codes for another receptor that defines both HSC and megakaryocyte identities. Our study shows that *Itga2b* expression is controlled by lineage-specific networks and associates with H4K8ac in megakaryocyte or H3K27me3 in the multipotential hematopoietic cell line HPC7. Correlating with the decrease in H3K27me3 at the *Itga2b* Iocus, we find that following commitment to megakaryocyte differentiation, the H3K27 demethylase Jmjd3 up-regulation influences both *Itga2b* and *Mpl* expression.

## Introduction

Our understanding of the transcriptional regulation of gene expression has been considerably expanded as knowledge of the role of epigenetic modifications has become clearer. It is now apparent that the mechanisms that create and maintain a permissive or repressive epigenetic environment play a pivotal part in gene regulation controlling developmental and cellular differentiation programs. Studies focusing on the histone modifications underlying transcriptional regulation suggest that acetylation promotes transcriptional activity, perhaps by maintaining an open chromatin state [1], while methylation of a variety of histone residues has been linked with either silencing or activating functions [2], [3]. However, these generalisations do not reflect entirely the complexity of the epigenetic regulation of transcription. In fact, the same modification can be associated with different outcomes depending on the context. This has been exemplified in embryonic stem (ES) cells by the presence of widespread ‘bivalent’ domains in which positive (H3K4me3) and negative (H3K27me3) histone modifications coexist on developmentally important genes in a ‘poised’ state [4]. Although crucial for the orchestration of gene expression during embryonic development [5], these bivalent domains cannot alone account for the simultaneous activation and repression of multiple genes that is essential for controlling developmental and differentiation processes and they must work dynamically in concert with other mechanisms. Such changing patterns of histone modifications are brought about by a set of enzymes including histone acetyl transferases (HAT), histone deacetylases (HDAC), methylase transferases, and demethylases [6].

Identifying emerging definitive HPC and HSC in the embryo proper, *Itga2b*/CD41 expression is influenced by developmental mechanisms [7] [8], [9]. The emergence of the first definitive HSC coincides with the appearance of intra-aortic clusters of CD41+ hematopoietic cells connected to the endothelial cells forming the ventral wall of the dorsal aorta. Recently, the model in which mesodermal cells produce a hemogenic endothelium capable of generating HSC has been reinforced by several studies [10]–[11], thus substantiating the fact that HSC and endothelial cells are developmentally related. Molecular changes accompanying this developmental process might therefore be inferred from the comparison of the two lineages downstream of the hemangioblast. Studies employing cell lines have proven very useful in assessing similarities and specificities of genome-wide regulatory mechanisms between different lineages. For example, the ENCODE project, based on six different human cell lines, has hugely improved the understanding of epigenetic regulation [12]. Although the lack of a human cell line model for the first definitive HSC has prevented such analysis, murine ES cells, transduced with the LIM-homeodomain protein Lhx2, have been used to generate CD41+ early hematopoietic stem/progenitor (HPC) cell lines, such as HPC7 [13]. The ectopic expression of Lhx2 was shown not to alter HSC identity and function as Lhx2-immortalised bone marrow HSC retain repopulation capacity in lethally irradiated recipient mice [13]. The HPC7 line presents the characteristics of the first definitive HSC [14], and together with endothelial cells (EC) and ES cells, constitutes a powerful framework for studying this critical developmental stage at the molecular level.

Here, we characterise the epigenetic environment of the *Itga2b* locus in these cellular models, mimicking different stages relative to hemangioblast emergence and commitment, and compare our findings to human data available from the ENCODE project [12]. We also draw a parallel with *Mpl* gene regulation because, like *Itga2b,* it is; 1) a marker of HSC [15], playing a role in the earliest stages of HSC development [16], and 2) up regulated during megakarypoiesis. Although very dissimilar phenotypically, megakaryocytes display many similarities with HSC and are also closely related to hemangioblasts [17]. Beside the surface receptors CD41 and c-Mpl, HSC and megakaryocytes also share signalling molecules and critical transcription factors [18]. Among the latter, the Ets and Gata families of transcription factors have essential roles that contribute to both cellular identities, raising the possibility that related regulatory networks are active in HSC and megakaryocytes [18]. Known targets for Ets- and Gata-mediated regulation in megakaryocytes, *Itga2b* and *Mpl* transcriptional control could exemplify such common sub-networks. To probe this hypothesis, the HPC7 line represents an ideal cellular system because of its unique capacity to recapitulating the differentiation process *in vitro* and produce normal mature megakaryocytes in response to thrombopoietin (TPO) [13].

Our study points to a crucial role for internal alternative promoters in the silencing of *itga2b* and *Mpl* expression in non-hematopoietic cells. Together with the switch in promoter usage, we highlight the profound restructuring of histone modification that needs to take place during development to enable the expression of the surface receptors in HSC. We show that *Itga2b* is then regulated by a HSC-specific transcriptional network that associates with a defined epigenetic landscape. Upon commitment to megakaryocyte differentiation, we find that the up-regulation of the H3K27me3 demethylase Jmjd3 plays a determining role in enabling the transition from HPC- to megakaryocyte-associated expression of both *itga2b* and *Mpl.*

## Results

### The *Itga2b* Epigenetic Landscape Varies during Development

**Figure 1:**
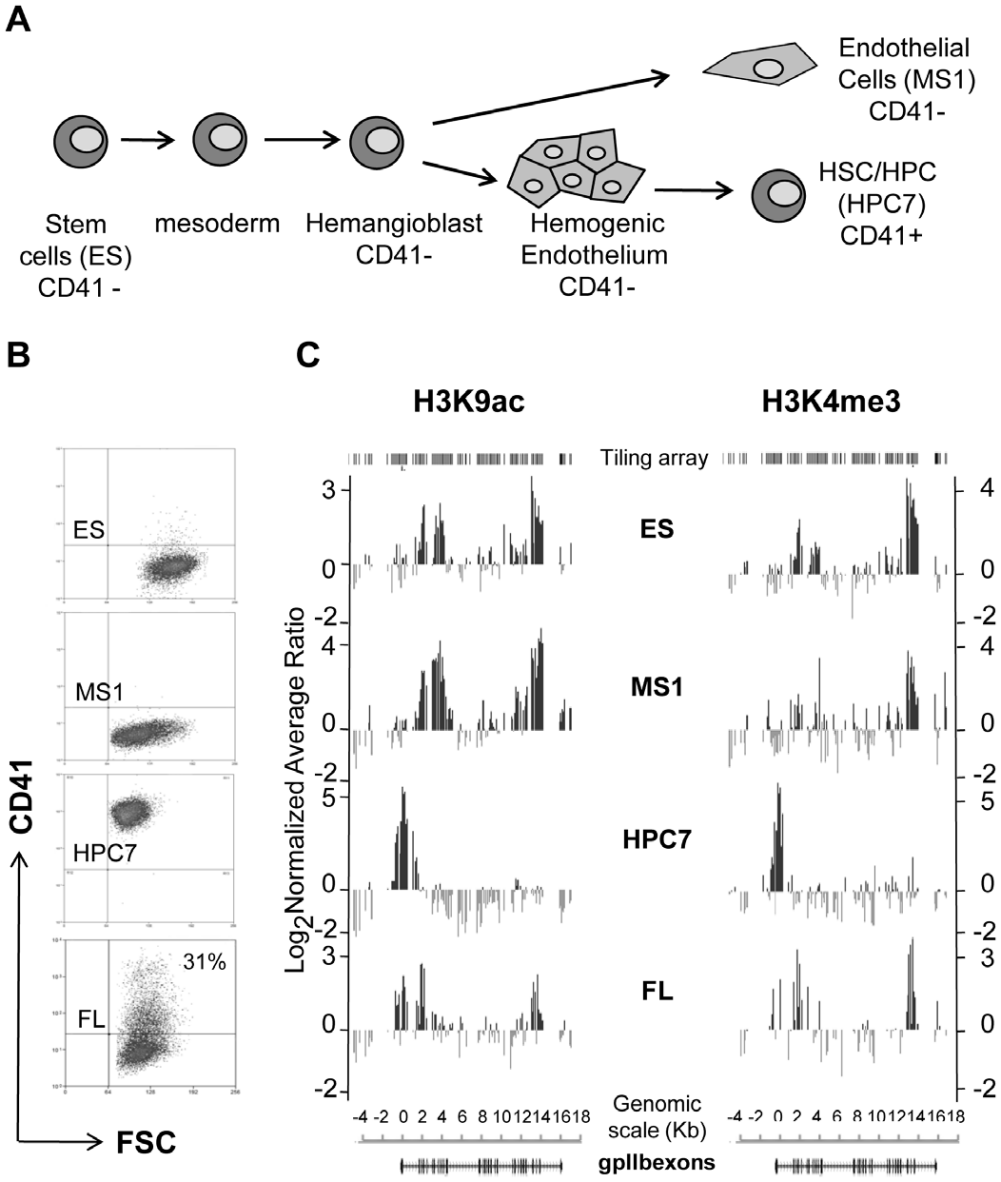
Epigenetic regulation of the *Itga2b* locus at the onset of hematopoiesis. (A) Schematic diagram of hematopoietic stem cell development. (B) ChIP on chip profiles of histone modifications across the Itga2b locus and CD41 expression in an HSC-like line (HPC7), E11.5 fetal liver cells (FL), EC (MS-1) and ES cells. ChIP assays were performed with anti-H3K9ac and anti-H3K4me3. Fold enrichments are plotted (log_2_) against genomic position in kilobases (kb). The position of the oligonucleotides spotted on the array (tiling array) and the *Itga2b* transcript are represented on the upper panels.

In order to approach the transcriptional regulation of the *Itga2b* gene at the onset of haematopoiesis, we compared cell systems modelling different cellular contexts prior to and subsequent to haemangioblast commitment (Figure 1A). We used ES cells as model for early embryonic cells and compared them to the ES-derived hematopoietic stem/progenitor HPC7 line and to the MS1 endothelial line. The expression of CD41 for the different cell lines and foetal liver cells from E11.5 embryos was tested by flow cytometry (Figure 1B right panel). This analysis showed that the ES and endothelial cells (MS1) are negative for this marker whereas the HPC7 is a uniform population of CD41+ cells. Moreover, with approximately 30% of CD41 expressing cells, the E11.5 foetal liver proved to be a rich source of primary CD41+ HSC and progenitors. The different cell populations were used for assessing transcriptional activity-associated histone marks by ChIP on chip experiments with antibodies against H3K9ac and H3K4me3 (Figure 1B left panel). The distribution of both modifications largely overlapped in CD41- cell types examined and differs from that of the CD41+ HPC7 cells. In HPC7 the two histone marks were exclusively associated with the *Itga2b* transcription start site (TSS). In contrast, regions within the core of the gene, between positions +2.5 to +3.5 kb and +12 to +14 kb from the *Itga2b* ATG, showed higher levels of modification in ES and MS1 lines. Finally, the heterogeneous foetal liver cells population recapitulates precisely the mix of both patterns, confirming the presence of the two types of profile in primary cells. The presence of transcription-associated histone modifications at the *Itga2b* locus suggests that in ES and MS1 lines, the gene is either transcriptionally active or bare marks reminiscent of a developmental priming of the gene.

### The *Itga2b* and *Mpl* Loci Exhibit Internal Alternative Promoters

**Figure 2:**
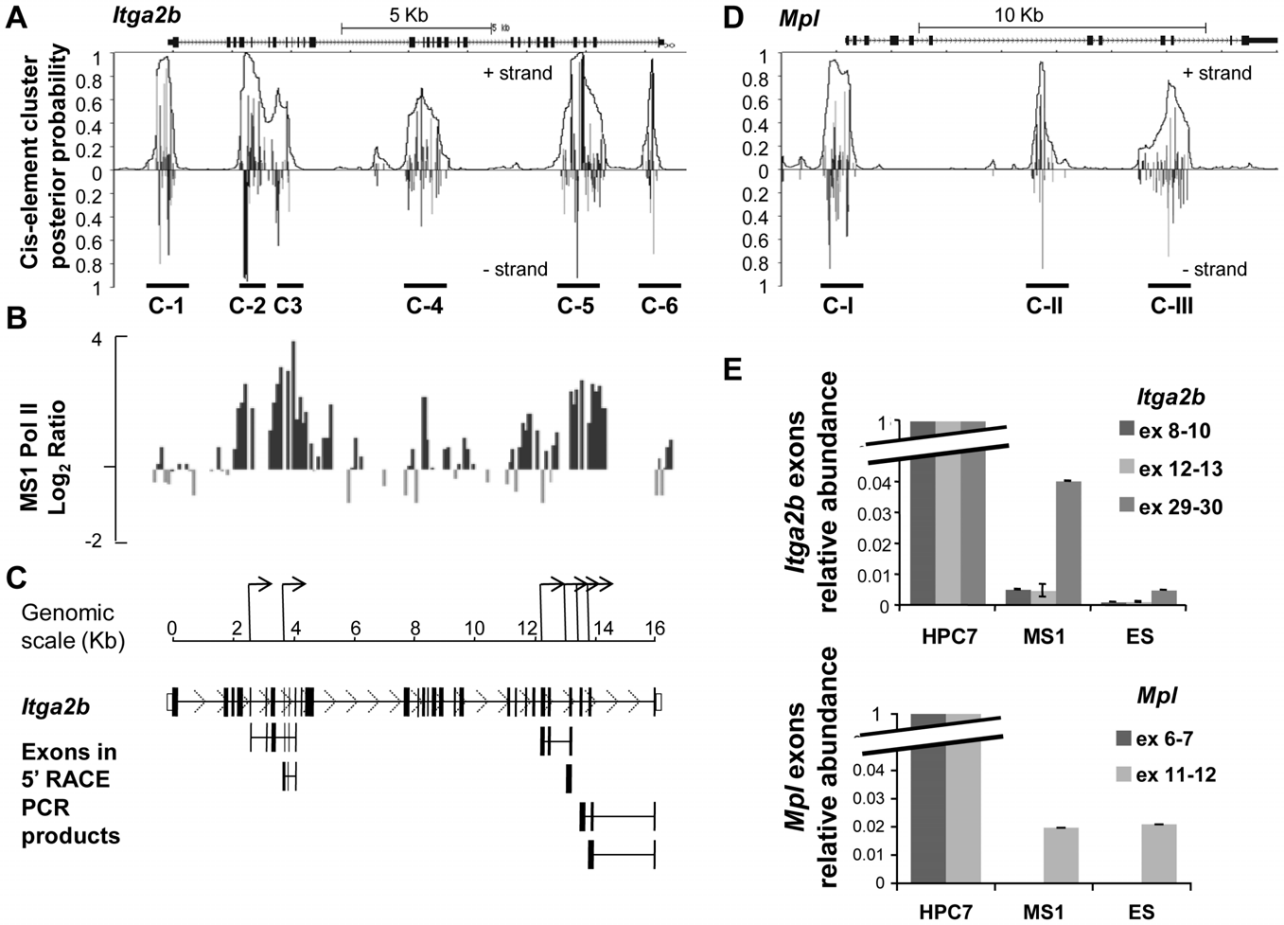
Transcriptional activity at the *Itga2b* locus in CD41- cells. (A) Cis-element cluster analysis at the *Itga2b* locus. Vertical lines indicate probabilities that regulatory factors bind to cis-elements at these positions. The overlain curve indicates the overall probability of being within a cluster of cis-elements bound by their factors. (B) RNA polymerase II (PolII) recruitment on the *Itga2b* locus in endothelial cells (MS1). Antibody against PolII was used to perform ChIP on chip. The fold enrichments are plotted on a logarithmic scale against the position across the *Itga2b* locus and aligned to the cluster predictive plot. (C) Schematic representation of the alternative *Itga2b* TSS deduced from 5¢ RACE PCR in MS1 cells. The lower panel indicates the full-length *Itga2b* exons and the exons cloned by RACE PCR (full sequences available in Figure S3). (D) Cis-element cluster analysis at the *Mpl* locus. (E) Relative levels of transcription assayed by Q-PCR for *Itga2b* exons 8–9–10, 11–12 and 29–30 and for *Mpl* exons 6–7 and 11–12 in MS1 and ES, and HPC7 cells. PCR results were normalized to HPRT and compared to the level measured in HPC7 cells**.** Error bars reflect standard error or the mean (SEM).

Often paired with promoter activity, the presence of both H3K9Ac and H3K4me3 within the *Itga2b* gene could indicate the existence of internal promoters. To substantiate this hypothesis, we searched for other promoter-like features. A search for clusters of transcription factor consensus binding sites highlighted the presence of six regions (C-1 to C-6, Figure 2A). Interestingly, whilst cluster C-1 is located at the normal *Itga2b* promoter, clusters C-2/C-3 and C-5 correlate with the position at which we detected H3K9ac and H3K4me3 modifications (Figure 1B). We next performed a ChIP on chip experiment on the endothelial line MS1 using an antibody against RNA polymerase II (PolII) to test for possible transcriptional initiation from these regions. The resulting profile (Figure 2B) demonstrated a broad accumulation of Pol II corresponding to the clusters C-2, C-3 and C-5, consistent with the opportunity for RNA expression from alternative promoters. We next cloned and sequenced the 5′ ends of the transcripts by RACE PCR using MS1 cell RNA (Figure 2C) and *Itga2b* specific primers (Table S1); this identified several TSS that correlate with the presence of PolII. With the possibility that the expression of alternative RNA could participate in the developmental regulation of a gene locus by preventing full-length transcript expression, we questioned whether a similar mechanism could regulate the *Mpl* gene, which codes for another surface receptor highly relevant to the first HSC [19]–[20]. The search for cluster of transcription factor consensus binding sites revealed the main *Mpl* proximal promoter identified by the cluster C-I and the presence of two potential internal promoters C-II and C-III (Figure 2D). We tested the expression of different exons from both *Itga2b* and *Mpl* loci in ES and MS1 lines by Q-PCR and assessed their level by comparison to the HPC7 cells. This analysis revealed a weak representation of exons situated downstream of the *Itga2b* and *Mpl* internal promoters (Figure 2E).

### Regulation of the *MPL* Locus Resembles that of the *ITGA2B* Gene

**Figure 3:**
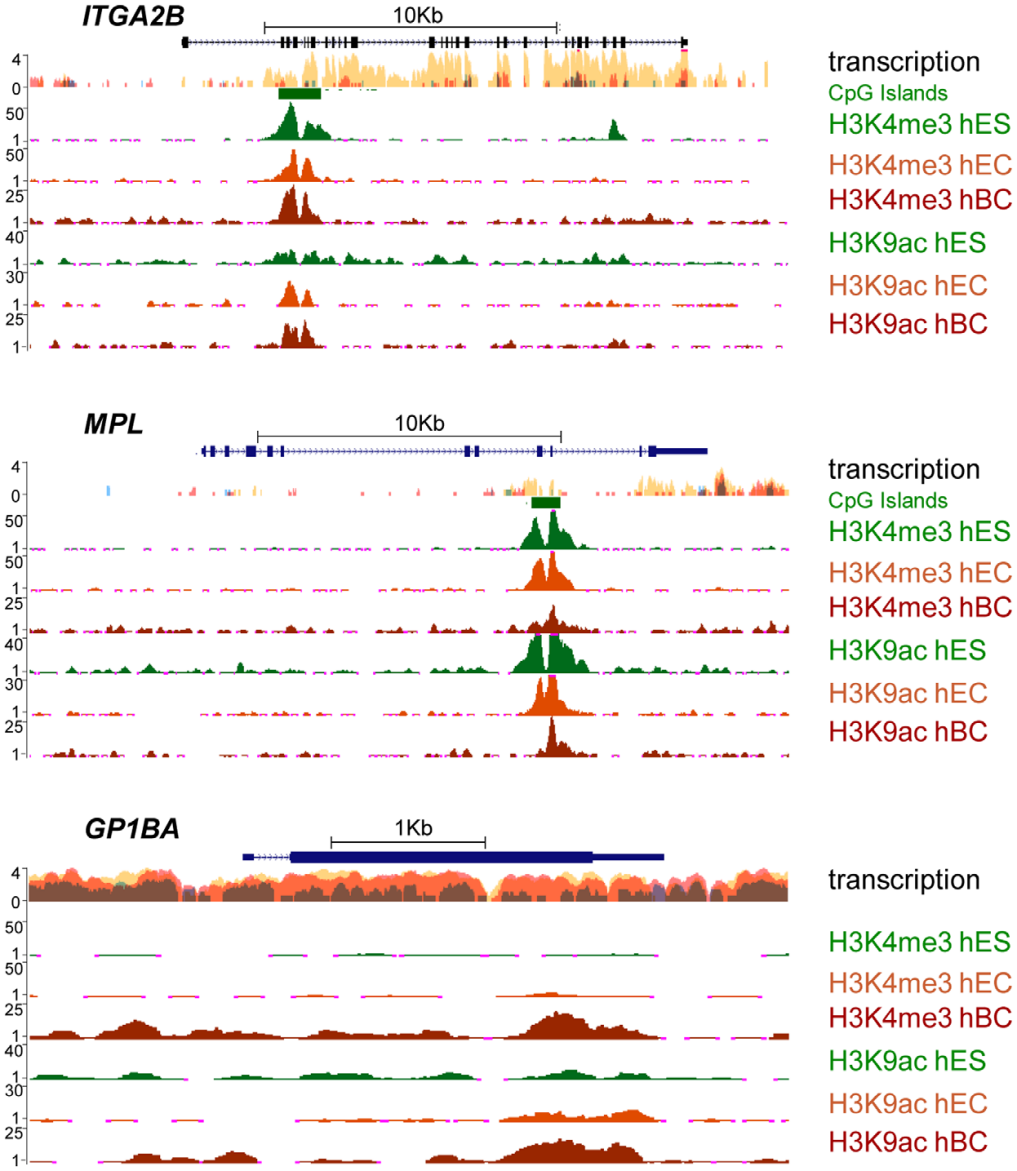
Encode project: RNA-seq and ChIp-seq data from CD41- cells. (A) Transcription levels assayed by RNA-seq and histone modification (H3K4me3 and H3K9ac) ChIP-seq on *ITGA2B, MPL,* and *GPIBA* in ES (H1-hESC), EC (HUVEC) and B lymphocytic (GM12878) cells. Plots are aligned to the transcript representations. CpG Islands are indicated by green boxes.

To cross-correlate our findings and further compare the regulatory features of *ITGA2B* and *MPL* loci in human cells, we made use of the publicly available ENCODE/Broad Institute project data [12]. Although, the project does not include a model for HSC, histone modification ChIP-seq profiles and RNA-seq data could be screened for human ES cells (H1-hESC, hES), endothelial cells (HUVEC, hEC line) and differentiated B lymphocytes (GM12878, hBC line). As previously, we chose to focus on H3K4me3 and H3K9ac as marks likely to associate with promoter activity, and compared their respective distribution profiles. This comparison was broadened to include the *GPIBA* locus to exemplify another early megakaryocyte marker although with no functional relevance to HSC. The resulting plots highlighted the association of H3K4me3 and H3K9ac with CpG islands within the core of the *ITGA2B* gene in hEC and hES cells (Figure 3A). Moreover, indicative of a promoter activity, the RNA-seq experiments revealed the expression of *ITGA2B* exons 5 to 30 in hES cells. Remarkably, the global comparison between the data associated with each gene revealed striking similarities between *ITGA2B* and *MPL*, both at the epigenetic and transcription levels. CpG islands, located in the vicinity of *MPL* exons 9–10, associate with the expression of the last four exons in human ES cells. Undefined within the *GPIBA* locus, such features differ from those of *ITGA2B* and *MPL* in their consistency across cell types. We note that H3K4me3 and H3K9ac mark the same potential promoters in the differentiated hematopoietic cells (hBC cells), suggesting that such positioning is dynamic and not fixed through developmental process.

### Different Sub-networks Regulate *Itga2b* in CD41+ HPC and Megakaryocytes

**Figure 4:**
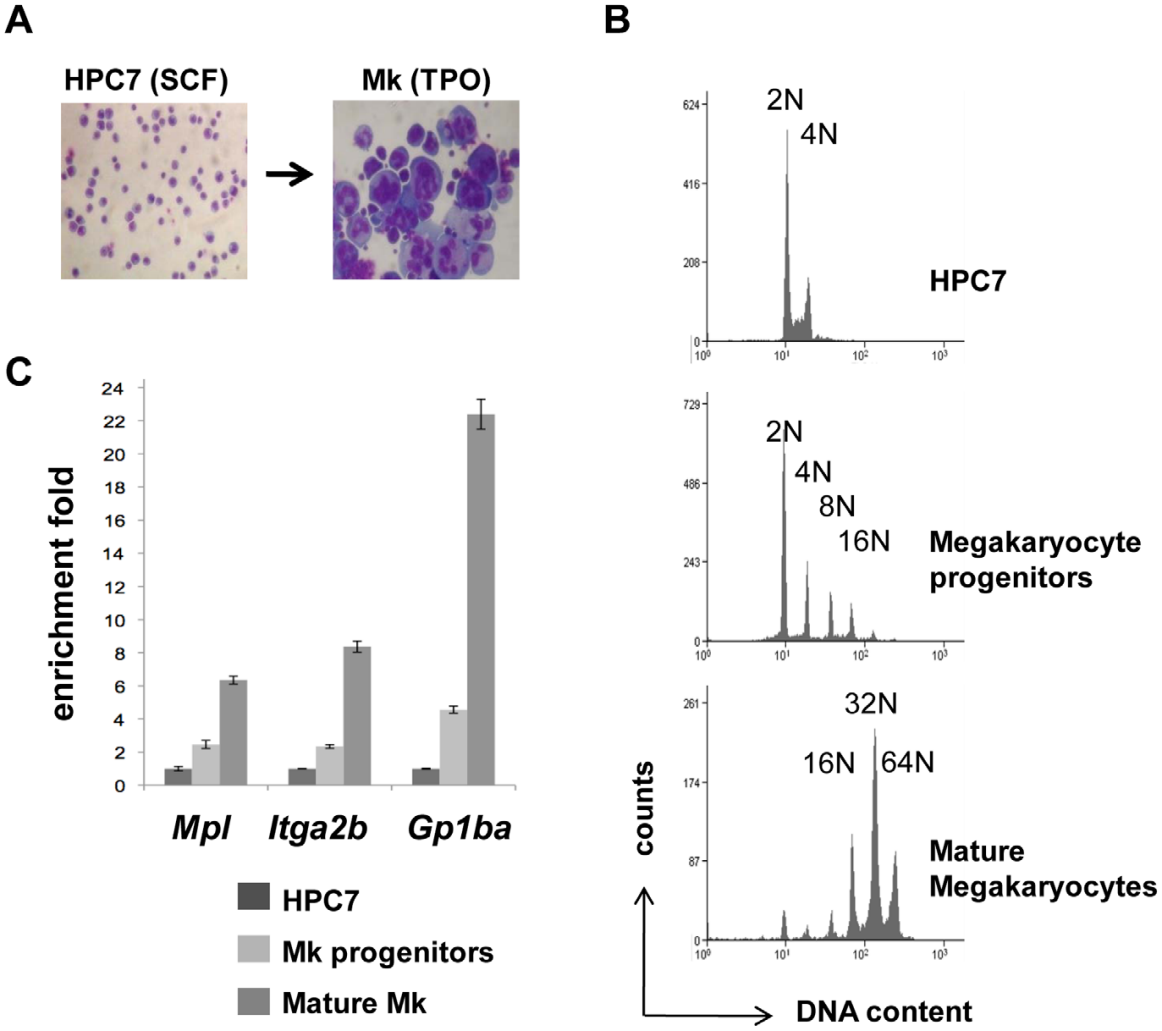
Generation of mature megakaryocytes through HPC7 in vitro differentiation. (A) Giemsa staining of HPC7 cells and HPC7-derived megakaryocytes. (B) Differentiating cells were separated by density gradient into populations at different maturation stages which levels of ploidy were assessed by flow cytometry in the presence of propidium iodide. (C) Levels of expression of *Itga2b* and *Mpl* were measured in each cell fraction by Q-PCR, normalized to the *GAPDH* PCR results and standardized to the HPC7 transcript level. Results, confirmed in three different experiments, were used to determine the two-tailed p-values by equal variance t-test. Indications of p values: *<0.05, and **<0.01.

**Figure 5:**
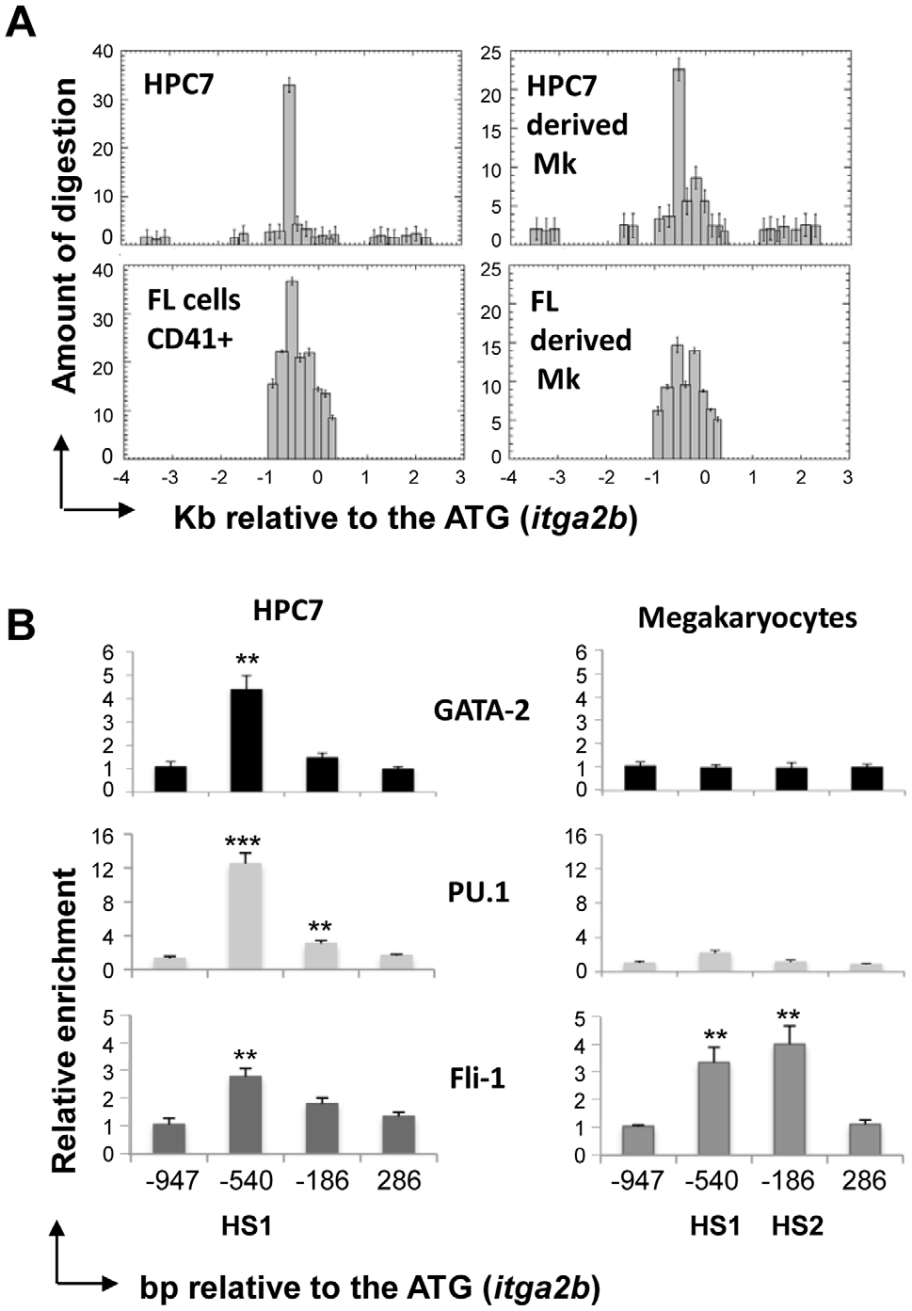
Detection of nuclease hypersensitive sites and transcription factor binding at the *Itga2b* promoter in CD41+ HPC and megakaryocytes. (A) DNA content analysis of undifferentiated HPC7 cells and HPC7-derived megakaryocytes. Mature megakaryocytes were separated by density gradient and ploidy was assessed by flow cytometry in the presence of propidium iodide. (B) Detection of nuclease hypersensitives sites. Nuclei from the HPC7 cells, HPC7-derived megakaryocytes, sorted AGM CD41+ cells and FL-derived megakaryocytes were digested using 60 units of DNaseI. The corresponding DNA were analysed by Q-PCR and compared to untreated samples to determine the amount of digestion. (C) Q-PCR analysis of X-Chip material for GATA-2, PU.1 and Fli-1 in HPC7 and megakaryocytes at the HS locations and at two surrounding control locations. Enrichments are given in comparison to the IgG control ChIP. Statistical significance: ***<0.001 and **<0.01. All results were confirmed in three independent experiments.

While it is clear that *Itga2b* gene regulation should dramatically differ in CD41+ HSC/HPC compared to CD41- non-hematopoietic cells, possible common regulatory features between the CD41+ HSC/HPC and megakaryocytes have never been assessed. To test this hypothesis, we generated megakaryocytes through TPO-dependent differentiation of the multipotential hematopoietic HPC7 line (Figure 4A). Cells at different stages of maturation were isolated by density-gradient fractionation and the differentiation process was assessed on the basis of DNA content, measured by flow cytometry, and mRNA expression. The lower density cells define a population of progenitors with zero to a few endoreplication cycles, whilst mature megakaryocytes with a typical median ploidy of 32N form the denser population (Figure 4B). Together with the HPC7 line, these cells were used to monitor the expression *Itga2b*, *Mpl* and *Gp1ba*. Indicative of normal megakaryopoiesis, all three markers were found up regulated in maturing cells (Figure 4C). To locate potential functional cis-regulatory elements in the *Itga2b* gene, nuclease hypersensitivity assays were performed on both undifferentiated and mature cells (Figure 5A). DNA from undigested and DNaseI-treated chromatin were analysed by Q-PCR across the *Itga2b* promoter and first exon. Reflecting the loss of template, the ratio of amplifications highlighted the presence of a nuclease hypersensitive site (HS) common to HPC7 cells and megakaryocytes (HS1) and a megakaryocyte-specific site (HS2). Although displaying different sensitivities, the HS locations were confirmed in primary CD41+ fetal liver cells and fetal liver-derived megakaryocytes (Figure 5A, lower panel). Both HS regions were found to encompass Ets and Gata binding sites that are conserved among species (Figure S1). Within these two transcription factor families, we chose to focus on Gata-2, Pu.1 and Fli-1 as candidate regulators of *Itga2b* in hematopoietic stem/progenitor cells and assessed their in vivo binding to the *Itga2b* promoter in both cell types. X-ChIP revealed that all three factors associate with HS1 in HPC7 cells (Figure 5B). In differentiated megakaryocytes, the absence of binding of Gata-2 and Pu.1 is consistent with the previous finding that these factors are repressed during the last stages of megakaryocytic maturation [21]. The concomitant up-regulation of Fli-1 appears to associate with its recruitment to both HS1 and HS2 regions, mirroring the changes in hypersensitive sites seen between HPC7 and megakaryocytes (Figure 5A). Altogether, these results show that distinct transcriptional sub-circuits control *itga2b* expression in HPC and megakaryocytes.

### Specific Epigenetic Features at the *Itga2b* Promoter Associate with the Level of CD41 Expression

**Figure 6:**
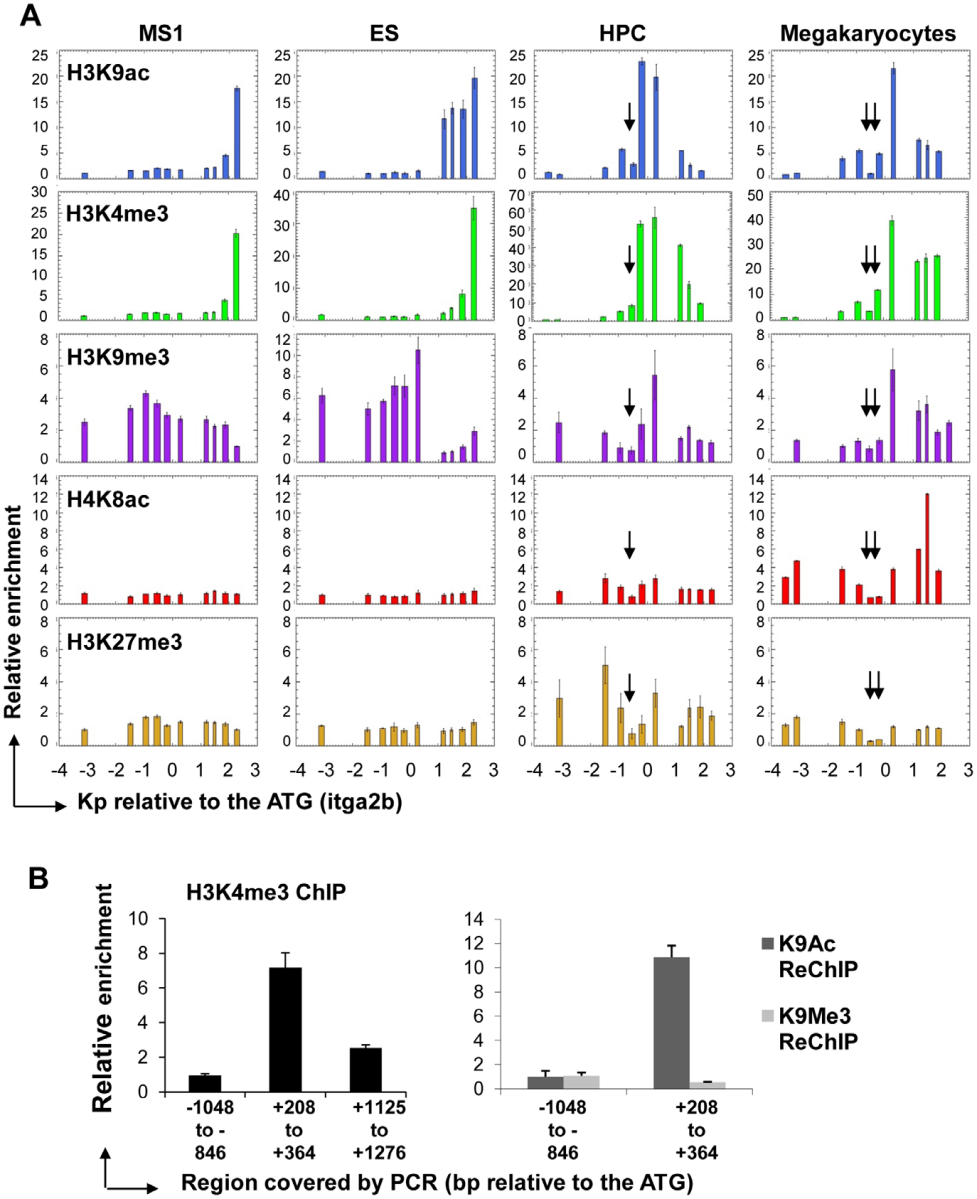
Histone mark distribution around the *Itga2b* promoter in EC, ES cells, HPC and mature megakaryocytes. (A) ChIP was performed using antibodies against H3K9ac, H3K4me3, H3K9me3, H4K8ac and H3K27me3 histone marks. ChiP material were analyzed by Q-PCR and levels of enrichment determined against the control IgG ChIP. (B) Temporal exclusion of the H3K9me3 modification with H3K4me3 and H3K9ac. Sequential ChIP experiments were performed with first a H3K4me3 antibody followed by reChIP using either a H3K9ac or H3K9me3 antibody. The pull down of H3K4me3-associated chromatin was verified by measure of enrichments against the initial input. Maximal enrichment, between +208 and +364 bp from the ATG is represented normalised to the upstream region −1048 to −846 bp (right panel). Equal amount of H3K4me3 ChIP chromatin material were used as inputs for ReChIP experiments with H3K9ac and H3K9me3 specific antibodies. The relative enrichments were determined against the input and normalized against the background as measured across the upstream region −1048 to −846 bp from the ATG. All profiles are representative of 3 independent experiments. Error bars reflect standard error or the mean (SEM).

We further tested the specificity of *Itga2b* transcriptional regulation in the CD41+ HPC7 compared to CD41+ megakaryocytes and CD41- ES and endothelial MS1 cells by assessing the occurrence of acetylated histones H3 and H4 (H3K9ac or H4K8Ac) and methylated histone H3 (H3K4me3, H3K9me3 and H3K27me3) around the TSS for the full-length transcript which we will refer to as main TSS (Figure 6A). Consistent with nucleosomal loss associated with the presence of functional cis-regulatory elements, all profiles for HPC7 and megakaryocytes displayed low levels of enrichment at the sites of nuclease hypersensitivity (Figure 6A, arrows). In addition, the H3K4me3 and H3K9ac profiles proved to be consistent with our previous observations: both modifications were detected immediately downstream of the main TSS in the CD41+ cells, but associated with the downstream internal promoter in CD41- cells. In the latter populations, the presence of H3K9me3, but not H3K27me3, upstream of the *Itga2b* main TSS suggests a possible role for H3K9me3 in transcriptional repression at the main promoter. Surprisingly, some levels of H3K9 tri-methylation were also detected, together with the H3K4me3 modification, downstream of the *Itga2b* main TSS in CD41+ HPC7 and megakaryocytes. We tested the possibility of a physical co-existence of these three modifications by performing sequential ChIP experiments with HPC7 chromatin using first an H3K4me3 antibody followed by reChIP with either an H3K9me3 or an H3K9ac antibody (Figure 6B). The region carrying H3K4me3 proved to be enriched for H3K9ac but not for H3K9me3. Hence, the observed co-location may reflect a mixture of two temporally exclusive situations. Importantly, by comparing HPC7 and megakaryocytes, we observed two major epigenetic differences over the *itga2b* promoter region (Figure 6A). First, a low level of H4K8ac in HPC7 contrasts with a strong peak of acetylation downstream of the *Itga2b* main TSS in megakaryocytes. Second, H3K27 tri-methylation was only observed at the *Itga2b* locus in HPC7 cells.

Overall, our analysis indicates that specific epigenetic landscapes associate with the different levels of *Itga2b* expression. While the locations of H3K4me3 and H3K9ac closely relate to CD41 expression, lack of acetylation combined with H3K27 tri-methylation appear to correlate with a lower level of expression of *Itga2b* in HPC7 and could reflect the requirement for tighter transcription regulation of the locus in hematopoietic stem/progenitor cells compared to megakaryocytes.

### Jmjd3-dependent Regulation of *Itga2b* and *Mpl* in Maturing Megakaryocytes

**Figure 7:**
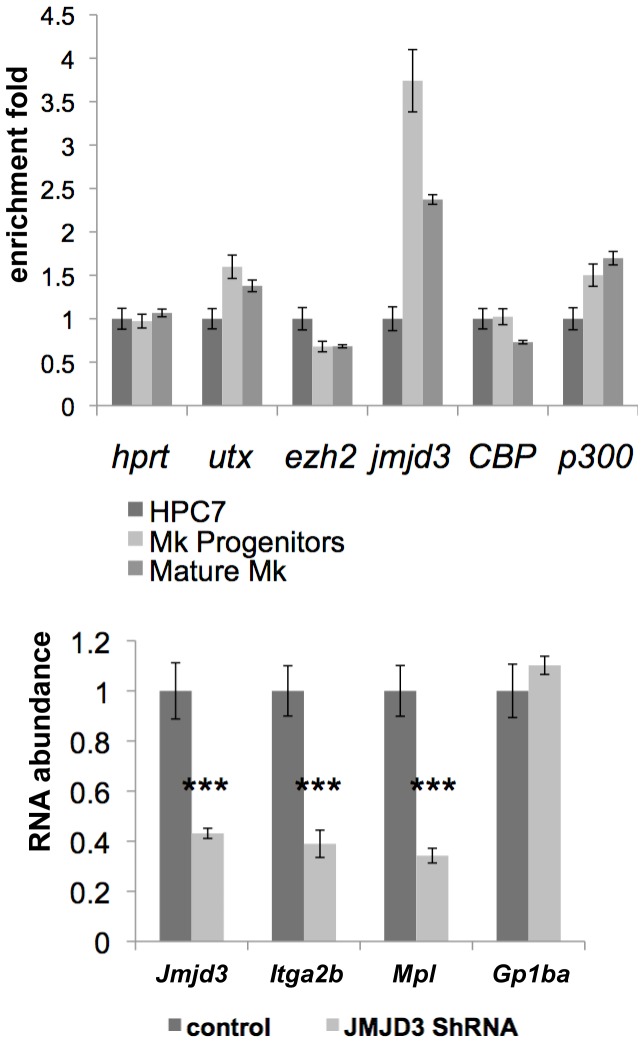
*Jmjd3* up-regulation affects *Itga2b* and *Mpl* expression during megakaryopoiesis. (A) Measure of expression of the histone modifiers *Utx*, *Jmjd3*, *Ezh2*, *CBP* and *p300* in HPC, megakaryocyte progenitors and mature cells. The levels of expression were determined by Q-PCR, normalized to HPRT and the two megakaryocytic populations were standardized to the HPC7 results. (B) JMJD3 silencing in committed cells. HPC7 cells were transfected with Jmjd3 ShRNA or control vector. After 24hours, SCF was removed from the medium and replaced by TPO to promote megakaryocytic differentiation. Puromycin was added at 48hours and cells were harvested at 72hours after transfection for cDNA preparations. The level of expression of *Jmjd3*, *Itga2b*, *Mpl* and *Gp1ba* were assessed by Q-PCR and standardized to HPRT (primers sequence provided in Table S3). Results were confirmed by 4 experiments. The two-tailed p value was determined by paired t-test. Statistical significance: ***<0.001.

Changes in histone-related epigenetic modifications are the result of the action of specific enzymes. To test whether the megakaryocytic differentiation process influences their expression, we monitored the mRNA level of the H3K27-specific methyltransferase (Ezh2) and demethylases (Utx and Jmjd3) as well as co-effectors possessing acetyltransferase activity (CBP and p300) at different stages of maturation. As previously, HPC7-derived megakaryocytes were separated by density gradient fractionation into populations representing different levels of maturation. cDNA were generated from each cell population and the expression of *Utx*, *Jmjd3*, *Ezh2*, *CBP* and *p300* mRNAs was assessed by Q-PCR (Figure 7A). The results showed an increase in *jmjd*3 expression commitment with the loss of H3K27me3 at the *Itga2b* locus (Figure 6) and an increase in *Itga2b* RNA expression (Figure 4). To test further the role of Jmjd3 in the implementation of the megakaryocyte-specific regulation of the expression of markers common to megakaryocytes and HSC (*Itga2b*/CD41 and c-Mpl) and the megakaryocytic marker (GPIbá), we used shRNA-mediated silencing to impair *jmjd*3 up-regulation during megakaryocytic commitment and differentiation. HPC7 cells were transfected with jmjd3 shRNA and induced to differentiate towards the megakaryocytic lineage in the presence of TPO. Maturing cells were harvested at day 2 of the differentiation process to assess *Jmjd*3 expression as well as the levels of the *Itga2b*, *Mpl* and *Gp1ba* transcripts. Although *Jmjd3* knockdown did not entirely overcome Jmjd3 up-regulation at this stage of differentiation, it correlated closely with a significant inhibition of *Itga2b* and *Mpl* transcription (Figure 7B). As an internal control, the lack of effect on *Gp1b*a expression suggested that *Jmjd3* silencing did not affect the differentiation process *per se* and that the decreased levels of *Itga2b* and *Mpl* was not due to delayed maturation (Figure 7B). We conclude that the up-regulation of the H3K27 demethylase Jmjd3 during megakaryopoiesis participates in the transition in expression of *Itga2b* and *Mpl* from HSC/HPC- to megakaryocyte-associated levels.

## Discussion

The regulation of gene transcription during development and differentiation is a coordinated process involving controls at many levels. Illustrated by a rapid acquisition of CD41 expression upon ES cell commitment to differentiation towards hematopoietic cells, this mechanism needs to be very dynamic. Our analysis points to a pivotal role for epigenetic modifications associated with promoter activity regulation in the mechanisms leading to cell-type specific gene expression. We find that both the *Itga2b* and *Mpl* genes exhibit alternative promoters and that a switch in promoter usage needs to take place during development to permit CD41 and c-Mpl expression at the surface of the emerging HSC. Multiple promoter usage is an important gene regulatory mechanism, the global significance of which has been highlighted by comprehensive analysis of genomes showing that 30 to 50% of mouse and human genes utilise more than one promoter [5], [22], [23]. Alternative promoters can be associated with germinal cell development, embryonic development, tissue-specific gene expression and differentiation processes [24]. Controlled by two distant promoters, the regulation of the master regulator of hematopoiesis Runx1 exemplifies an aspect of such phenomena [25], [26].

Here we describe a mechanism in which, prior to the specification of the HSC, accessibility to the *Itga2b* promoter is reduced through epigenetic means, whereas internal initiation of transcription may contribute in silencing CD41 expression. In CD41- ES and endothelial cells, the absence of the H3K27me3 Polycomb-associated modification indicate that the repression of CD41 expression is mediated through a Polycomb-independent mechanism. In effect, the observed patterns suggest that the presence of H3K9me3 over the main TSS region of the *Itga2b* gene, combined with the absence of marks associated with transcriptional activity, could be responsible for inhibiting the main promoter activity. In this setting, transcription initiation may occur from internal alternative promoters containing the active gene associated modifications H3K4me3 and H3K9ac. Further inquiries of the ENCODE/Broad Institute project data [12] confirm the recruitment of the basic transcriptional machinery in both human and murine ES cells (Figure S2). JunD, TBP and USF2 are found associated to the *ITGA2b* and *MPL* alternative promoters in human ES cells, while p300 and PolII positioning correlate with DNaseI hypersensitivity sites in regions we had previously defined in murine ES cells. As the first definitive HSC emerge, the acquisition of CD41 expression implies an epigenetic remodelling to produce the landscape that is permissive for the transcription of the full-length transcript, as observed in the CD41+ hematopoietic stem/progenitor HPC7 line. The relative importance of such a mechanism, compared to the well-described bivalent marks [4]–[5], remains to be investigated. However, the fact that we could find parallel features on the *Mpl* locus suggests that similar mechanisms could regulate both genes and possibly a class of HSC-related genes.

The dissociation of transcriptional activity and protein expression resulting from RNA transcription could serve diverse purposes such as priming genes for expression by maintaining them within transcription factories or silencing genes that are retained in the transcription factories as a result of adjacent actively transcribed loci. In fact, ubiquitously expressed genes flank both *Itga2b* and *Mpl* loci. Furthermore, the reinstatement of H3K4me3 and H3K9ac at the internal locations upon HSC commitment to the B cell lineage suggests that the alternative promoters serve a repressive rather than priming function on CD41 expression. In contrast, CD41 and Mpl expression are up regulated during megakaryocyte differentiation.

Previous studies have identified Ets and Gata factors as key regulators for both genes in the maturation process [27] and the shared importance of these families of factors in HSC and megakaryocytes led to speculations regarding possible common transcriptional sub-networks [18]. Here we show that the identities of the family members, and therefore the composition of the activating network controlling CD41 expression is cell-type specific. At the epigenetic level, we have highlighted a pivotal role of histone modifications in the mechanisms leading to cell-type specific expression. A megakaryocyte-specific feature of *Itga2b* regulation is a high degree of acetylation on H4K8. Changes in histone acetylation, and the association with specific HAT and HDAC, are well documented in relation to transcriptional regulation during differentiation [28]. The CBP/p300 family of HAT are known to interact with various hematopoietic transcription factors including Pu.1, Gata-1, and Gata-2, which are substrates for their activity [29], [30], [31], [32]. We have shown that within the hematopoietic compartment, the binding of Gata-2 and PU.1 to the *Itga2b* promoter is restricted to the uncommitted cells. Interestingly, the potential of Pu.1 to inhibit CBP-mediated acetylation of histones suggests that cross-regulation between HAT and Pu.1 possibly participates in the maintenance of the HPC-related epigenetic profile and level of transcription of the *Itga2b* gene. Moreover, Gata-1, which is specifically up regulated during megakaryopoiesis, can induce cell-type specific histone acetylation following its own acetylation [33]. Therefore, during megakaryopoiesis, changes in the transcriptional network, reflected in *Itga2b cis*-regulatory domain occupancy, would ultimately facilitate histone acetylation and the implementation of the H4K8ac lineage-specific pattern on the *Itga2b* promoter.

More surprisingly, we have defined an HPC-related epigenetic pattern on *Itga2b* implying a pivotal role for H3K27me3. Although H3K27me3 is a defined mark of polycomb-mediated repression of transcription, there is no strict correlation between H3K27me3 and transcriptional silence as it is also found in ES cells associated with transcribed loci such as wnt, fgf and hedgehog [34]. Our analysis of the *Itga2b* locus provides another example of a low level of H3K27me3 being compatible with transcriptional activity. Here we find that the up-regulation of the H3K27-specific demethylase Jmjd3 during megakaryopoiesis participates in implementing a permissive context for *Itga2b* and *Mpl* lineage-specific expression. The role of Jmjd3 in the transcriptional program associated with early stage commitment [35], [36] and differentiation processes [33], [37] is just beginning to be uncovered. However, the mode of action of Jmjd3 remains somewhat obscure as it appears to either rely on, or be independent of, its ability to demethylate its substrate [33], [36], [38]. We have not been able to detect Jmjd3 binding to the *Itga2b* locus following megakaryocyte lineage commitment; however, the lower levels of H3K27me3 across the *Itga2b* promoter in mature megakaryocytes suggest that *Itga2b* is a likely target for Jmjd3 demethylase activity.

Overall, we have demonstrated that the regulation of *Itga2b* expression involves a broad range of mechanisms. We show that these are closely linked to epigenetic modulation and are regulated in a developmental stage and differentiation-associated manner.

## Materials and Methods

### Cell Culture and Differentiation

HPC7 cells were kindly provided by Dr Lief Carlsson, and were cultured in Stem Pro 34 medium (Invitrogen) and recombinant SCF 100 ng/ml. To induce megakaryocytic differentiation, the SCF concentration was lowered to 20 ng/ml and recombinant TPO (Peprotech) was added to a concentration of 100 ng/ml for two days, than the SCF was removed whilst the TPO remained for an additional 5 days. Mature megakaryocytes were purified by density gradient as previously described [21].

### Cell Phenotype and DNA Content Analysis

Cells were cytospun and stained using Diff-Quik reagents as specified by the manufacturer (Dade Behring, Atterbury Milton Keynes, UK). DNA content was determined by staining with 50 µg/mL propidium iodide (Sigma-Aldrich, Gillingham, UK) as previously described [39]. Cell-cycle analysis was performed with a FACScan analyzer and Summit software (Becton-Dickinson).

### X-ChIP and Antibodies

Cross-linking and X-ChIP were performed as previously described [40]. Most of the antibodies against histone modifications were made in-house as previously described [41] with the exception of antibodies against H3K27me3 (Millipore). All antibodies against transcription factors were purchased from Santa Cruz Biotechnology, Inc.

### ChIP on Chip Analysis

A series of 60 base-long oligonucleotides were designed to span the *Itga2b* locus and compared against the mouse genome using BlastN to avoid repeated or cross-reacting sequences. The oligonucleotides were arrayed in triplicate onto Codelink slides (Amersham GE healthcare, Little Chalfont, UK) using a Microgrid II arrayer (Biorobotics/Genomic Solutions, Cambridge, UK) and stored at room temperature until hybridised. Samples were hybridized as previously described [42] and fold enrichments over input were normalized to the median of values across the locus.

### Cis-element Cluster Finder

Clustering of transcription factor consensus binding sites was assessed using the web-based Cister algorithm [43], [44] (http://zlab.bu.edu/~mfrith/cister.shtml) using the default parameters: 35 bp average distance between motifs within a cluster, average number of 50 motifs in a cluster, average distance between clusters of 2 kb. The motif probability threshold was set to 0.01.

### Race PCR

Rapid amplifications of cDNA were achieved using the 5′/3′RACE kit 2^nd^ generation (Roche). The different primers used for first strand synthesis and nested PCR are listed in Table S1.

### Nuclease Hypersensitive Site Mapping

Cells were washed with PBS and nuclei prepared by resuspension in 1 ml aliquots of digestion buffer (Tris-HCl 15 mM pH7.5, NaCl 15 mM, KCl 60 mM, MgCl_2_ 5 mM, glucose 300 mM, EGTA 0.5 mM, NP40 0.1%). For digestion of nuclei, 0 to 100units of DNaseI were added to each aliquot and incubated 10 minutes at 37°C. The reaction was terminated by adding 330 µl of stop solution (EDTA 100 mM, SDS 4%). RNA and proteins were sequentially digested by addition of 100 µg of RNase A and 100 µg of proteinase K, incubated respectively for 1 hour and overnight at 37°C. Following phenol/chloroform extractions, DNA was ethanol precipitated and resuspended in water. Note that when treating small amounts of nuclei, all volumes were halved. Quantitative PCR was performed on 30 ng of undigested and DNaseI-treated DNA. The different sets of *Itga2b* oligonucleotides used are listed in Table S2.

### Transfection and Plasmids

20×10^6^ HPC7 cells were co-electroporated with a plasmid conferring puromycin resistance (1 µg) and the Jmjd3 shRNA or control vectors (5 µg) (Origene, Cambridge, UK), using the Amaxa transfection kit (Biosystems, Warrington, UK) according to the manufacturer’s instructions. Puromycin (Invitrogen) was added to the medium to a final concentration of 0.5 µg/ml, 24 hours post transfection.

## Supporting Information

Figure S1 Multi-species sequence alignment of the Itga2b nuclease hypersensitive regions. Boxes indicate the locations of Ets and Gata conserved consensus binding sites.
(TIFF)

Figure S2 Encode project ChIP-seq data from CD41- cells. (A) Transcription factors binding on *ITGA2b* and *MPL* loci in human ES cells. (B) DNaseI hypersensitivity, p300 and pol II binding on *Itga2b* and *Mpl* in murine ES cells (ES-Bruce4) and bone marrow cells.
(TIFF)

Figure S3 RACE PCR sequences.
(DOC)

Table S1 5′ RACE PCR primers sequences.
(DOC)

Table S2 Sequences and positions of the Q-PCR primers spanning the *Itga2b* locus.
(DOC)

Table S3 Sequences of Q-PCR primers.
(DOC)
