# Supplementary figures and images for: Itga2b Regulation at the Onset of Definitive Hematopoiesis and Commitment to Differentiation
Source: PLoS One. 2012 Aug 28;7(8):e43300. doi: 10.1371/journal.pone.0043300 (PMC3429474; doi:10.1371/journal.pone.0043300)

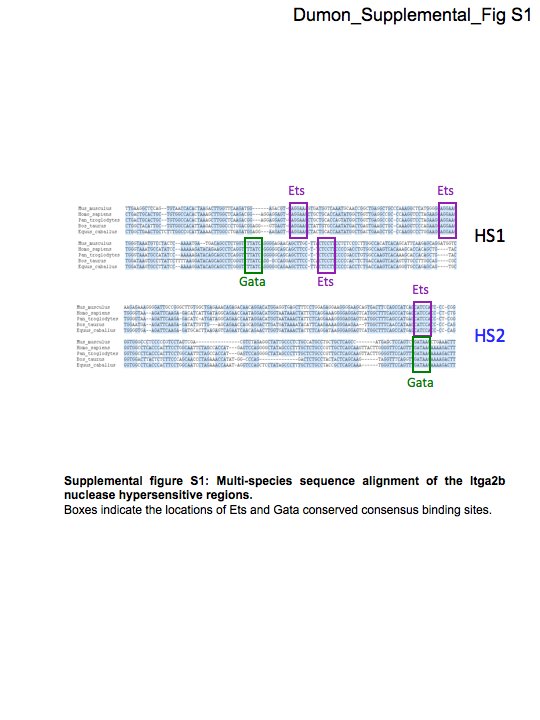

Supplement: Figure S1 — Multi-species sequence alignment of the Itga2b nuclease hypersensitive regions. Boxes indicate the locations of Ets and Gata conserved consensus binding sites. (TIFF) [file pone.0043300.s001.tiff]

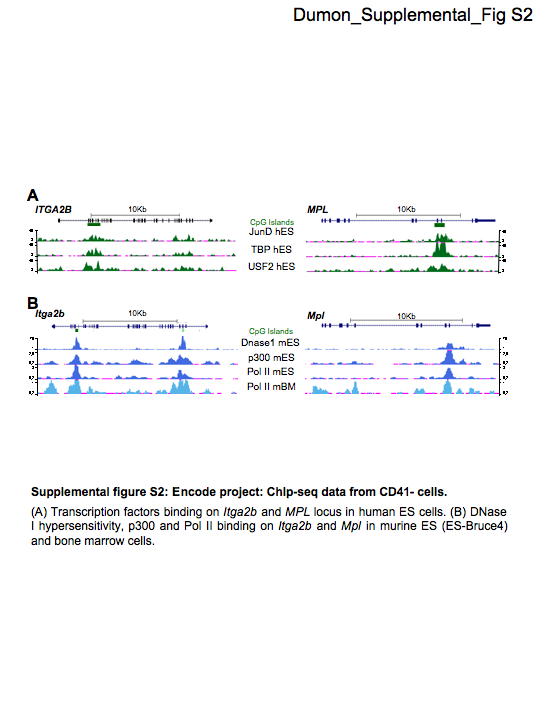

Supplement: Figure S2 — Encode project ChIP-seq data from CD41- cells. (A) Transcription factors binding on ITGA2b and MPL loci in human ES cells. (B) DNaseI hypersensitivity, p300 and pol II binding on Itga2b and Mpl in murine ES cells (ES-Bruce4) and bone marrow cells. (TIFF) [file pone.0043300.s002.tif]
