# Supplementary material for: Itga2b Regulation at the Onset of Definitive Hematopoiesis and Commitment to Differentiation
Source: PLoS One. 2012 Aug 28;7(8):e43300. doi: 10.1371/journal.pone.0043300 (PMC3429474; doi:10.1371/journal.pone.0043300)
Supplement: Figure S3 — RACE PCR sequences. (DOC) [file pone.0043300.s003.doc]

**Figure S3: RACE PCR sequences**

E1, TSS +3582 bp from *Itga2b* ATG (intron7/8, exon8, exon9, exon10)

TTCTTCCCCACACATGTCSACTTCCCATCCACRCTGGYTCACCCTTCTCGCTGKGGATTCTAGTGARATCTTGGGCACGCCTCAKTTTTCCTASCCSCCCTAGGCAKTTTATACTAATGACCTCGGTACYCCTGATCCTTGATCTTTTCCTTCTTGCTTTACTCGRAAACTGCTAAACTGTGCTGTGTCTTCAGGATATTCGGTAGCCGKGGGCRAKTTTGATGGGGATCCGASCMCTACARAGTACKTATCGGGTGCCCCCACTTGGAGCTGGACCTTGGGAGCGGTGGAAATTTTGGACTCYTACTACCAKCCC

E2, TSS +3666 bp from *Itga2b* ATG (intron7/8, exon8, exon9, Exon10)

GTTTTCCTAGCCSCCCTAGGCAGTTTATACTAATGACCTCGGTACTCCTGACCCTTGATYTTTTCCTTCTTGCTTTACTCGGAAACTGCTAAACTGKGCTGTGTCTTCAGGATATTCGGTAGCCGKGGGCGAGTTTGATGGGGATCCGAGCACTACARAGTACGTATCGGGKGCCCCCACTTGGAGCTGGACCTTGGGAGCGGKGGAAATTTTGGACTCCTACTACCAGCCC

E3, TSS 3673 bp from *Itga2b* ATG (intron7/8, exon8, exon9, Exon10)

TAGCCSCCCTAGGCAGTTTATACTAATGACCTCGGTACTCCTGACCCTTGATYTTTTCCTTCTTGYTTTACTCGGAAACTGCTAAACTGTGCTGTGTCTTCAGGATATTCGGTAGCCGKGGGCGAGTTTGATGGGGATCCGAGCACTACAGAGTACGTATCGGGKGCCCCCACTTGGAGCTGGACCTTGGGAGCGGKGGAAATTTTGGACTCCTACTACCAGCCC

E4, TSS +3666 bp from *Itga2b* ATG (intron7/8, exon8, exon9, Exon10)

GTTTTCCTAGCCSCCCTAGGCAGTTTATACTAATGACCTCGGTACTCCTGACCCTTGATYTTTTCCTTYTTGCTTTACTCGGAAACTGCTAAACTGKGCTGKGTCTTCAGGATATTCGGTAGCCGKGGGSGAGTTTGATGGGGATCCGAGCACTACAGAGTACGTATCGGGKGCCCCCMCTTGGAGCTGGACCTTGGGAGCGGKGGAAATTTTGGACTCCTACTACCAGCCC

E5, TSS +2602 bp from *Itga2b* ATG (intron4/5, exon5, exon6, exon7, exon8, exon9, exon10)

TCTTGTAGGCGGAGACAAGCGATACTGTGAAGCAGGCTTCAGCTTGGCGGTGACCCAGGCTGGGGAGCTAGTGCTTGGAGCCCCYGGAGGCTACTTTTTTTTAGGTCTCCTGGCTCGCGTTCCAATTGAGAACATCATCTCCAGCTACCGCCCGGGTACCCCTTTGTGGCATGTTTCCAACCAGCGCTTCACCTACGACAACAGCAACCCAGTGTTTTCCGATGGTTACCGGGGATATTCGGTAGCCGTGGGCGAGTTTGATGGGGATCCGAGCACTACAGAGTACGTATCGGGTGCCCCCACTTGGAGCTGGACCTTGGGAGCGGTGGAAATTTTGGACTCCTACTCCAGCCC

E6, TSS +2602 bp from *Itga2b* ATG (intron4/5, exon5, exon6, exon7, exon8, exon9, exon10)

TCTTGTAGGCGGARACAAGCGATACKGKGAAGCAGGYTTCAGCTKGGSGGKGACCCAGGCTGGGGAGCTAGKGCTKGGASCCCCTGGAGGCTACTTTTTTTTAGGTCTCCTGGCTCGCGTTCCAWTTGARAACATCATCTCCAGCTACCGCCCGGGTACCCTTTTGKGGCATGTTTCCAACCAGCGCTTCACCTACRACAACAGCAACCCAGKGTTTTCCRAKGGTTACCGGGRATATTCGGTAACCGKGGGCGAGTTTGAKGGGGATCCGAGCACTACARAGTACGTATCGGGKGCCCCCACTTGGAGCTGGACCTTGGGAGCGGKGGAAATTTTGGACTCCTACTACCAGCCC

E7, TSS +3666 bp from *Itga2b* ATG (intron7/8, exon8, exon9, exon10)

GTTTTCCTAGCCGCCCTAGGSAGTTTATACTAATGACCTCGGTACTCCTGACCCTTGATYTTTTCCTTYTTGCTTTACTCGGAAACTGCTAAACTGKGCTGKGTCTTCAGGATATTCGGTAGCCGKGGGCGAGTTTGATGGGGATCCGAGCACTACAGARTACGTATCGGGKGCCCCCMCTTGGAGCTGGACCTTGGGAGCGGKGGAAATTTTGGACTCCTACTACCMSCCC

DW1, TSS +12418 bp from *Itga2b* ATG (exon25, exon26, exon27)

CTCAGACTTCTCATCCACATTCCTGGCCAGTCCCAGCCCTCGGATCTGCTCTACATCCTGGATGTGCAGCCGCAGGGAGGTCTCCTTTGCTCCACACAGCCATCTCCCAAGGTGGACTGGAAACTATCCACGCCCAGCCCTTCTTCCATTCGCCCCGTCCATCACCAGCGTGAGCGCAGACAGGCATTCCTGCAGGGGCCCAAGCCAGGGCAGCAGGACCCAGTTCTGGTGAGCTGCGACGGCTCAGCGTCCTGTACGGTGGTGGAGTGTGAGCTG

DW2, TSS +12386 bp from *Itga2b* ATG (exon25, exon26, exon27)

TCCACAACATTGGCCCTGGCACTGTGAATGGCCTCAGACTTCTCATCCACATTCCTGGCCAGTCCCAGCCCTCGGATCTGCTCTACATCCTGGATGTGCAGCCGCAGGGAGGTCTCCTTTGCTCCACACAGCCATCTCCCAAGGTGGACTGGAAACTATCCACGCCCAGCCCTTCTTCCATTCGCCCCGTCCATCACCAGCGTGAGCGCAGACAGGCATTCCTGCAGGGGCCCAAGCCAGGGCAGCAGGACCCAGTTCTGGTGAGCTGCGACGGCTCAGCGTCCTGTACGGTGGTGGAGTGTGAGCTG

DW3, TSS +12463 bp from *Itga2b* ATG (exon25, exon26, exon27)

CTGCTCTACATCCTGGATGTGCAGCCGCAGGGAGGTCTCCTTTGCTCCACACAGCCATCTCCCAAGGTGGACTGGAAACTATCCACGCCCAGCCCTTCTTCCATTCGCCCCGTCCATCACCAGCGTGAGCGCAGACAGGCATTCCTGCAGGGGCCCAAGCCAGGGCAGCAGGACCCAGTTCTGGTGAGCTGCGACGGCTCAGCGTCCTGTACGGTGGTGGAGTGTGAGCTG

DW4, TSS +13247 bp from *Itga2b* ATG (intron26/27, exon27)

GAAGCTCATCCAGAGACCAGTGCTTCCTGACCCTCTGGCTCTCCGCGTTGGGTCCTTGACTGATGGGAGGGATGATTGGCAGGAGGGCCTGTGAACTTACCACACATCCATCCCCCTCAGAGCTGCGACGGCTCAGCGTCCTGTACGGTGGTGGAGTGTGAGCTG

DW5, TSS +13249 bp from *Itga2b* ATG (intron26/27, exon27)

AGCTCATCCAGAGACCAGTGCTTCCTGACCCTCTGGCTCTCCGCGTTGGGTCCTTGACTGATGGGAGGGATGATTGGCAGGAGGGCCTGTGAACTTACCACACATCCATCCCCCTCAGAGCTGCGACGGCTCAGCGTCCTGTACGGTGGTGGAGTGTGAGCTG

DW6, TSS +12517 bp from *Itga2b* ATG (exon25, exon26, exon27)

CCATCTCCCAAGGTGGACTGGAAACTATCCACGCCCAGCCCTTCTTCCATTCGCCCCGTCCATCACCAGCGTGAGCGCAGACAGGCATTCCTGCAGGGGCCCAAGCCAGGGCAGCAGGACCCAGTTCTGGTGAGCTGCGACGGCTCAGCGTCCTGTACGGTGGTGGAGTGTGAGCTG

D1, TSS +13938 bp from *Itga2b* ATG (intron28/29, exon29, exon30)

TGTGGGACTGAGGTCTGCTTACTAGGAGCCGGCTTGGCTGAGCATCCTTCTCCGCGGTCACAGCCCCCCTGAAGTGACATCTAGTTTGCCCTCAGGTGCAGACACAGCTGCTTCGGGCCTTGGAGGAGAGGGCCATTCCTGTCTGGTGGGTGCTGGTGGGCGTGCTGGGCGGTCTGCTGCTGCTGACCCTGCTAGTTTTGGCCATGTGGAAGGCTGGCTTCTTCAAGCGGAATCGACCGCCTCTGGAGG

D2, TSS +13940 bp from *Itga2b* ATG (intron28/29, exon29, exon30)

TGGGACTGAGGTCTGCTTACTAGGAGCCGGCTTGGCTGAGCATCCTTCTCCGCGGTCACAGCCCCCCTGAAGTGACATCTAGTTTGCCCTCAGGTGCAGACACAGCTGCTTCGGGCCTTGGAGGAGAGGGCCATTCCTGTCTGGTGGGTGCTGGTGGGCGTGCTGGGCGGTCTGCTGCTGCTGACCCTGCTAGTTTTGGCCATGTGGAAGGCTGGCTTCTTCAAGCGGAATCGACCGCCTCTGGAGG

D3, TSS + 13668 bp from *Itga2b* ATG (intron27/28, exon28, exon29, exon30)

CCTCGATTAGCTGTGGTAAGCGCCTGTCACCCCCGCCCCATACAGAGGCCGCAGGAGCAGTTTGTGCTGCAGTCGCACGCCTGGTTCAACGTCTCCTCCCTACCTTACTCGGTGCCGGTGGTCAGCTTGCCCAGTGGGCAAGCTCGGGTGCAGACACAGCTGCTTCGGGCCTTGGAGGAGAGGGCCATTCCTGTCTGGTGGGTGCTGGTGGGCGTGCTGGGCGGTCTGCTGCTGCTGACCCTGCTAGTTTTGGCCATGTGGAAGGCTGGCTTCTTCAAGCGGAATCGACCGCCTCTGGAGG

D4, TSS +14048 bp from *Itga2b* ATG (exon29, exon30)

CTTCGGGCCTTGGAGGAGAGGGCCATTCCTGTCTGGTGGGTGCTGGTGGGCGTGCTGGGCGGTCTGCTGCTGCTGACCCTGCTAGTTTTGGCCATGTGGAAGGCTGGCTTCTTCAAGCGGAATCGACCGCCTCTGGAGG

D5, TSS +13542 bp from *Itga2b* ATG (intron27/28, exon28, exon29, exon30)

TTGCTTTGGGTTTGAGCGGGTTCACACAAGGCCTGGGGAAGATTTGCCTTGGGGCGGGGATAGGGTAAAACCCAGGCGGGACTCAACAGCAGGGAGCCCCGGGCTTGGATTGCGGCCATCTCCAGGCCTCGATTAGCTGTGGTAAGCGCCTGTCACCCCCGCCCCATACAGAGGCCGCAGGAGCAGTTTGTGCTGCAGTCGCACGCCTGGTTCAACGTCTCCTCCCTACCTTACTCGGTGCCGGTGGTCAGCTTGCCCAGTGGGCAAGCTCGGGTGCAGACACAGCTGCTTCGGGCCTTGGAGGAGAGGGCCATTCCTGTCTGGTGGGTGCTGGTGGGCGTGCTGGGCGGTCTGCTGCTGCTGACCCTGCTAGTTTTGGCCATGTGGAAGGCTGGCTTCTTCAAGCGGAATCGACCGCCTCTGGAGG
