# Supplementary material for: Itga2b Regulation at the Onset of Definitive Hematopoiesis and Commitment to Differentiation
Source: PLoS One. 2012 Aug 28;7(8):e43300. doi: 10.1371/journal.pone.0043300 (PMC3429474; doi:10.1371/journal.pone.0043300)
Supplement: Table S1 — 5′ RACE PCR primers sequences. (DOC) [file pone.0043300.s004.doc]

Table TS1: 5’ RACE PCR primers sequences

|  | **Primers 5’→3’** | **Primers 5’→3’** | **Primers 5’→3’** |
| --- | --- | --- | --- |
| cDNA first strand synthesis | TGCAGACAAGCCTCTCAAAGC | ACTCTTCCTCTTCATCTTCCTCCAGA | ACCGGCACCGAGTAAGGTAGG |
| 1^st^ nested PCR | GGCAGCCACAGCAATATCATT | cctccagaggcggtcgattc | CACAAACTGCTCCTGCGGCCT |
| 2^nd^ nested PCR | GGGCTGGTAGTAGGAGTCCAAAA | Not needed | cagctcacactccaccaccg |
