# Supplementary material for: Itga2b Regulation at the Onset of Definitive Hematopoiesis and Commitment to Differentiation
Source: PLoS One. 2012 Aug 28;7(8):e43300. doi: 10.1371/journal.pone.0043300 (PMC3429474; doi:10.1371/journal.pone.0043300)
Supplement: Table S2 — Sequences and positions of the Q-PCR primers spanning the Itga2b locus. (DOC) [file pone.0043300.s005.doc]

**Table TS2:** **Sequences and positions of the Q-PCR primers spanning the *Itga2b* locus**

| **Amplicon**  **(bp from ATG)** | **Forward primers 5’→3’** | **Reverse primers 5’→3’** |
| --- | --- | --- |
| -3595 → -3354 | CTGCTGCCCCTGAGCCATCT | CGCCACACTAAGGAATTTGAACATC |
| -3206 → -3000 | CCAGTGTCAACCAGACAAAGCAA | AAGCCCAGGACACTCAGGACC |
| -1570 → -1393 | GAGTGGTAAAGTAGGATAAGGCAGTGG | GGCAAAGGGTCGTAGGACTATAGCCAC |
| -1048 → -846 | GATTCAGCCTTTCAGCAGCACTAC | AACTGTTTGTGGACGGAGTCACTG |
| -868 → -630 | AGTGACTCCGTCCACAAACAGTTC | TACACTGGAGCCTTCAATACTGGG |
| -653 → -427 | CCCAGTATTGAAGGCTCCAGTGTAAC | CCTGCTCTTGAATGCTGTGATGTG |
| -476 → -272 | TGCTTACTCCTTCCTCTCCCCTTG | GGCAATCCCCTTTCTCTTTCTCTTC |
| -299 → -72 | GAGGAAGAGAAAGAGAAAGGGGATTG | CGGAAGTTTCAGCTTATCAGACTGG |
| -96 → +67 | CCAGTCTGATAAGCTGAAACTTCCG | TAGGACCCAAGAACAGTGGTGTCC |
| +54 → +234 | GTTCTTGGGTCCTAGTGCTGTTCC | TTCGAGGGTGCAGACCTCTCTTA |
| +208 → +364 | GCGTTAAGAGAGGTCTGCACCCTC | TGTGCTCATCTCCCCATTGCTATC |
| +322 → +479 | AGGGTGGGTACTGGGGAGAGATAG | CAGGTAGGTACTTGTAGCTTTGCCAG |
| +1125 → +1276 | CTCCTGGCATCAGAGCTCCTGAG | CGGCAACATGCTTTTGTAAACGT |
| +1255 → +1435 | CGTTTACAAAAGCATGTTGCCGG | GGGAGAGCTCACTGGGATGTACG |
| +1439 → +1543 | CCTTCCCCCTTTCAACCAGGT | CGATTTCCCTCACCCTCACTCC |
| +1525 → +1724 | GTGAGGGTGAGGGAAATCGGTGT | CGTGGCATAGGAAAAACTGGACC |
| +1773 → +1963 | GGCCATAACCTGTGCTCATCCAG | GGACCCCCTTTCCCCTTCATT |
| +1961 → +2161 | TCCCCAGGTGGAAATGGATGG | CCCTACCCTGGATTTGCTCCC |
