# Supplementary material for: Itga2b Regulation at the Onset of Definitive Hematopoiesis and Commitment to Differentiation
Source: PLoS One. 2012 Aug 28;7(8):e43300. doi: 10.1371/journal.pone.0043300 (PMC3429474; doi:10.1371/journal.pone.0043300)
Supplement: Table S3 — Sequences of Q-PCR primers. (DOC) [file pone.0043300.s006.doc]

**Table TS3:** **Sequences of Q-PCR primers**

| **transcript** | **Forward primers 5’→3’** | **Reverse primers 5’→3’** |
| --- | --- | --- |
| Utx | ATCAGAATGGACATCCCACCC | GGCACCGTCAATGTGTTTCC |
| jmjD3 | CAGTCCACTTCCAACTCCATCTG | CCTTCTGCAACCAATTCCAGC |
| Ezh2 | AAAGACCCTGAATGCAGTCGC | CACATTCTCTGTCACCATGCACTT |
| HPRT | CCAGCGTCGTGATTAGCGATG | ATAGCCCCCCTTGAGCACACAGAG |
| CBP | AGGCAGGAGGCATGACCAA | TCCCACTGATGTTTGCAACTG |
| P300 | TCATGAACGGTTCCATTGGA | CTGAATTTGGAGACCAAGGCC |
| Itga2b | TTTCTGCAGCCTAAGGGCC | GGCAGCCACAGCAATATCATT |
| Gp1ba | AGCTAGTAGAGACAAGGACCGAGTCAT | AGGCTGGTCACTTTGGAGATACTACAA |
